# Exploring Concomitant Acetylcholinesterase Inhibitor and Overactive Bladder Anticholinergic Use and Risk of Hospitalization in Medicare and Dual-Eligible Medicare–Medicaid Populations in a Historic Database

**DOI:** 10.3390/pharmacy11050140

**Published:** 2023-09-07

**Authors:** Jonathan H. Watanabe, Tu Hoang

**Affiliations:** 1 Center for Data-Driven Drugs Research and Policy, School of Pharmacy & Pharmaceutical Sciences, University of California, Irvine, 856 Health Sciences Quad Drive, Suite 5400, Irvine, CA 92697, USA; 2 School of Pharmacy & Pharmaceutical Sciences, University of California, Irvine, 856 Health Sciences Rd., Irvine, CA 92697, USA; nnhoang2@hs.uci.edu

**Keywords:** acetylcholinesterase inhibitors, anticholinergics, antimuscarinics, overactive bladder, Alzheimer’s disease, dementia

## Abstract

Overactive bladder prevalence increases in older adults often complicating the management of other comorbidities. The theoretical antagonism between the parasympathetic-blocking anticholinergic agent and the parasympathetic stimulatory agents concomitantly used by patients is only recently being explored. The primary aim was to determine the frequency of the annual use of acetylcholinesterase inhibitors, overactive bladder anticholinergics, and the use of both agents in the same year. The secondary aim was measurement of the association between annual hospitalization and same-year use of both acetylcholinesterase inhibitors and anticholinergics. The US nationally representative MarketScan^®^ Medicare databases were analyzed. In the Medicare enrollees, there were 122 020, 141 920, and 15 639 users of acetylcholinesterase inhibitors, anticholinergics, and both agents, respectively. The percentage of acetylcholinesterase inhibitor users who also used anticholinergics was 12.8%. Comparing users of both acetylcholinesterase inhibitors and anticholinergics to those using AChEI alone, 5 608 of the former experienced a hospitalization (35.9%) compared to 33 182 of the latter (31.2%). There was an increased risk of hospitalization for those using both acetylcholinesterase inhibitors and anticholinergics in the same year, with an odds ratio (OR) of 1.23 (95% CI, 1.19, 1.28). Clinicians should consider improved monitoring of the usage of both medications and clarify alternative regimens that avoid anticholinergics in at-risk older adults.

## 1. Introduction

Overactive bladder (OAB) is a chronic medical condition defined by the International Continence Society (ICS) as group of symptoms consisting of urinary urgency, usually accompanied by increased daytime frequency and nocturia, with or without urgency urinary incontinence, in the absence of a urinary tract infection or other obvious pathology [1]. OAB is prevalent in more than ten percent of the global population and is expected to rapidly increase with the aging of the worldwide population [2]. In the US, it is estimated that the older population in 2030 will be twice as large as in 2000, growing from 35 million to 71.5 million and representing nearly 20 percent of the total U.S. population. The U.S Census Bureau projects that the portion of the population aged 85 and over could grow from 5.3 million in 2006 to nearly 21 million by 2050 [3]. With a prevalence in excess of 34 million people, OAB is one of the most common chronic conditions in the US, affecting more people than either diabetes or lower back pain [4,5,6]. OAB prevalence expands in older adults often complicating the management of other comorbidities common to older adults [7]. Some comorbidities that can cause or contribute to OAB in older adults are diabetes, neurological conditions, heart disease, sleep disorders, depression, and increased risk of falls and fractures [8].

Pharmacologic therapy for patients that is not adequately managed with lifestyle modifications commonly begins with the use of antimuscarinic medications, a type of anticholinergic agent [9]. These medications are one of the first-line treatments for OAB [10]. Anticholinergic agents function in OAB via blockade of muscarinic receptor activation and inhibit the detrusor muscle contractions found in an overactive bladder [11]. However, the anticholinergic activity commonly leads to an array of adverse events that includes dry mouth, dry eyes, confusion, constipation, somnolence, blurred vision, and increased heart rate [10,11].

On the opposing side of the pharmacologic spectrum to anticholinergics, acetylcholinesterase inhibitors (AChEIs) are a class of drugs that activate muscarinic receptors in the peripheral nervous system by mimicking the effects of acetylcholine (ACh) on the parasympathetic nervous system [12]. Motivated by the cholinergic hypothesis of Alzheimer’s disease, there have been multiple research attempts to develop treatments for Alzheimer’s disease that enhance the ability of the cholinergic effect to modulate synapses in the brain at least in the early stage of the disease [12,13]. These medications included acetylcholinesterase inhibitors that interrupt the breakdown of acetylcholine, which increases the binding of ACh to cholinergic receptors [14]. Currently, there are several FDA-approved medications that improve cholinergic function with the goal of managing Alzheimer’s disease. The list includes donepezil, rivastigmine, and galantamine that have each been shown to statistically significantly improve cognition and daily and global function for patients with Alzheimer’s disease.

The pharmacologically opposing functions of anticholinergics and acetylcholinesterase inhibitors translate to reduced healthy function for patients on concomitant therapy due to increased porosity of the blood–brain barrier (BBB) [15]. In this example of therapeutic competition, brain M1 muscarinic receptors are antagonized by OAB anticholinergic agents. The anticholinergic molecules for OAB management are derived from the ammonium molecule. Replacement of at least one hydrogen atom of ammonium with an aryl or alkyl group is required for the construction of OAB anticholinergic medications. This therapeutic family includes the OAB agents darifenacin, fesoterodine, oxybutynin, solifenacin, and tolterodine. These tertiary ammonium compounds exhibit neutral polarity and elevated lipophilicity which increase their ability to cross the BBB with darifenacin, oxybutynin, and solifenacin. These features significantly demonstrate higher levels of BBB penetration [16,17]. The presence of anticholinergic medication produces a drop in the functional levels of acetylcholine in the brain [18]. Acetylcholine is the major neurotransmitter in the cholinergic system. Over several decades, subsequent research studies have supported the fundamental role of the cholinergic system in memory mechanisms that relate to a neurodegenerative condition like Alzheimer’s disease [14]. Additionally, the severity of the condition was found to have a positive correlation with the level of cholinergic dysfunction that translated to learning and memory impairment. These findings led to the development of acetylcholinesterase inhibitors as pharmacological therapies for cognitive deficits in patients with Alzheimer’s disease [19].

The potential antagonistic pharmacology between the parasympathetic-blocking anticholinergic agent and the parasympathetic stimulatory agents used by patients with syndromes such as Alzheimer’s disease is only recently being explored in research [20]. A recent prospective study demonstrated that patients prescribed anticholinergics with the highest exposure based on total daily doses, including anticholinergics for treatment of OAB, were at increased risk for incident dementia and Alzheimer’s disease [21]. Compared to persons without dementia, persons with dementia were more likely to receive medications that would be categorized as medication misuse and overuse across a variety of domains. In a nationally representative study, the array of medications included commonly used medications for the treatment of hypertension and diabetes as well as medications that negatively affect cognition [22]. Another retrospective study examined the use of anticholinergics in patients with dementia versus matched controls. The study concluded that older patients with dementia were more likely to take anticholinergics than matched controls. Specifically, clinicians did not recognize the adverse effects of anticholinergics and the antagonism between these medications attributable to concomitant use of donepezil and anticholinergics [23]. While these studies demonstrated the use of anticholinergics in older patients with dementia, the studies did not evaluate if hospitalization risk was elevated for concurrent use of anticholinergics and acetylcholinesterase inhibitors.

The goals of this analysis were to quantify the proportion of acetylcholinesterase inhibitor users that also used an anticholinergic for overactive bladder management and to determine if users of both anticholinergics and AChEI were at increased risk of hospitalization compared to users of AChEI agents alone.

## 2. Materials and Methods

The primary aim was to use the US nationally representative MarketScan^®^ Medicare medication and inpatient databases to determine the frequency of the annual use of acetylcholinesterase inhibitors, anticholinergics for overactive bladder, and the use of both agents in the same year in enrollees of Medicare and, separately, dual-eligible Medicare–Medicaid enrollees (‘Duals’). The secondary aim was to determine the association between annual hospitalization and the same-year use of both AChEIs and AChs compared to users of AChEIs alone.

The MarketScan databases have served as the source of health services analyses for more than 300 peer-reviewed manuscripts. The data come from a selection of nationally representative large employers, health plans, and government and public organizations. The annual medical databases include private-sector health data from approximately 100 payers. Historically, more than 500 million claim records have been available in the MarketScan databases.

### 2.1. Patients

This database was specifically designed by the investigator to include Medicaid as well as Medicare enrollees. Moreover, indicators were requested to designate Medicaid enrollees who were eligible for Medicare. This served to define the ‘Duals’ category. The MarketScan Medicare Supplemental and Coordination of Benefits (COB) Database was created for Medicare-eligible retirees with employer-sponsored Medicare Supplemental plans. The MarketScan Medicaid Database contains the pooled healthcare experience of approximately seven million Medicaid enrollees from multiple states. This allows for the comprehensive assessment of low-income older adult enrollees.

### 2.2. Exposure and Outcomes Variables

The MarketScan outpatient medication claims file includes a therapeutic class variable for each medication used by all enrollees. Using the therapeutic class designation for each outpatient drug claim, categorical variables were created that indicate annual use of acetylcholinesterase inhibitor agents and anticholinergics for overactive bladder. These generated variables were then used to produce an indicator variable of annual use of both AChEIs and AChs. The hospitalization claims database was then applied to generate a yes/no dichotomous variable indicating the occurrence of at least one hospitalization in the same year as the medication use. Medicare eligibility is also afforded to those with end-stage renal disease as well as other syndromes and for some dependents of Medicare enrollees [24]. Since the population of interest in this investigation was older adults, only those 65 years old or above were included in this analysis.

### 2.3. Statistical Analysis

Frequency counts were executed to determine the frequency and proportion of medication therapeutic class use, the use of both classes of agents, and hospitalizations. Evidence of association between use of both agents and hospitalization was tested via chi-squared test. Logistic regression was used to quantify the risk of use of both agents and hospitalization compared to users of AChEIs alone. The dichotomous variable of annual hospitalization was used as the outcome variable (i.e., response variable) and the main effect variable (i.e., explanatory variable) for the regression model was the use of both AChEIs and AChs versus those who used AChEIs alone as the reference category. All analyses were completed using SAS 9.4 (Cary, NC, USA) with an α < 0.05. The University of California, Irvine Human Research Protections Program designated this research to be nonhuman subject research and thus exempt from institutional review board review.

## 3. Results

### Primary and Secondary Outcomes

In Medicare enrollees, there were 122,020, 141,920, and 15,639 users of acetylcholinesterase inhibitor, overactive bladder anticholinergics, and both agents, respectively. The percentage of AChEI users who also used an overactive bladder anticholinergic was 12.8%. Comparing users of both AChEIs and OAB AChs to those using AChEIs alone, 5608 of the former experienced a hospitalization (35.9%) compared to 33,182 of the latter (31.2%). There was a statistically significant association between use of both AChEIs and OAB AChs in the same year and hospitalization with a *p*-value < 0.001 (Table 1). Multiple logistic regression also demonstrated the association between annual use of both agents and elevated hospitalization. There was a statistically significant increased risk of hospitalization for those using both AChEIs and OAB AChs in the same year with an odds ratio (OR) of 1.23 (95% CI, 1.19, 1.28) (Table 2).

In the Duals, there were 8915, 7260, and 1160 users of an AChEI, overactive bladder anticholinergic, and both agents, respectively. The percentage of PS users who also used an overactive bladder anticholinergic in 2012 was 13.0%. Comparing users of both AChEIs and OAB AChs to those using AChEIs alone, 298 of the former experienced hospitalization (25.7%) compared to 1 804 of the latter (23.2%). While an increased percentage of those receiving both agents in the same year experienced a hospitalization the difference was not statistically significant in the Duals with a *p*-value of 0.07 (Table 3). There was a non-statistically significant increased odd of hospitalization for those receiving both agents with an OR of 1.14 (95% CI, 0.99, 1.31) (Table 2).

## 4. Discussion

With a rapidly aging global population, there is mounting concern about the use of antagonistic pharmacotherapies in the senior population. In both the Medicare and Medicare–Medicaid dual enrollees, roughly thirteen percent of those using an acetylcholinesterase inhibitor also consumed a mechanistically antagonistic anticholinergic for overactive bladder. In the Medicare enrollees, there was a statistically significant increased risk of hospitalization in the same year in the older adults using both AChEIs and OAB Achs versus those using AchEI alone. This is particularly concerning for patients and family/caregivers attempting to slow the path of disease progression and manage symptoms of Alzheimer’s disease by taking cholinesterase inhibitors such as donepezil, rivastigmine, and galantamine. Concomitant use of an anticholinergic in patients using a acetylcholinesterase inhibitor could translate to blunted symptom management or accelerate the path to dementia requiring hospitalization. This analysis suggests this may already be occurring.

While there was a crude increase in hospitalization for those using both agents in the Medicare–Medicaid dual-eligible population, the risk increase was not statistically significant. The improvement in the management of dual-eligible patients may be a potential explanation for this result of our study. The Duals’ health plan may also institute limits on the use of antagonistic therapies like the concurrent use of the two different medications in this study. Hence, Medicaid preferred drug lists and plan limits on the number of dispensed drugs that have these opposing features. However, even in this large nationally representative claims database, there were only 1 160 users of both acetylcholinesterase inhibitors and anticholinergics for overactive bladder in the Duals population compared to 15 639 users among Medicare enrollees. In future research efforts, it would be useful to conduct this analysis of the Duals in a pooled and enriched sample with the same main outcome to examine whether the relationship would achieve statistical significance in a larger powered study.

Predominantly via the inhibition of acetylcholinesterase enzymes, the consumption of acetylcholinesterase inhibitor medication stimulates the increase of synaptic levels of acetylcholine in the brain. These agents have been shown to improve functional status and neuropsychiatric outcomes in randomized and clinical trials [25]. A retrospective survival analysis examined the association of acetylcholinesterase inhibitors with mortality in a large cohorts of patients with Alzheimer’s disease from one of the largest healthcare providers in Europe in an evaluation for mental disorders and dementia. The study concluded that AChEIs medications were associated with a mortality reduced by more than 20% in adjusted statistical models [26]. However, a randomized pragmatic clinical trial study was conducted to compare the differences in adherence and adverse events of available AChEIs in a real-world clinical setting among new users of these medications. This study found that a high rate of adverse events presented a significant barrier to successful Alzheimer’s disease management [27].

Attenuation of cholinergic stimulation by acetylcholinesterase inhibitors due to anticholinergic blockade would be expected to worsen Alzheimer’s disease control. Recent analysis has demonstrated an elevated risk of incident dementia for subjects that used anticholinergics [28]. Clearly, an already present increased progression to dementia for anticholinergic users could be accelerated for those already taking acetylcholinesterase inhibitors.

Older adults are already more likely to have multiple health conditions which contribute to polypharmacy. It is also possible that a portion of the 10% of acetylcholinesterase inhibitor users that used anticholinergics that we observed in our study was attributable to a prescribing cascade phenomenon. Prescribing cascades typically begin when an adverse reaction to a drug is considered as a new medical condition. A new drug is prescribed for the mistaken diagnosis which multiplies the risk of developing additional adverse effects related to this unnecessary treatment [29]. A nationally representative study examined the medication misuse in community-dwelling persons with dementia in the US. Compared to individuals without dementia, patients with dementia are 41% more likely to be prescribed medications that negatively affect cognition that included strongly anticholinergic and sedative-hypnotic agents [22]. Another nationally representative study in older adults found that a majority of people with dementia expressed willingness to stop their medication if a clinician stated it was possible and the belief that at least one of their medications may be unnecessary [30]. We suggest healthcare providers such as physicians and pharmacists should monitor more closely to interrupt prescribing cascades and implement deprescribing interventions when it is necessary to improve quality of life for this vulnerable population.

There were limitations to this analysis. This analysis used a nationally representative medical claims database. Thus, this research was conducted in an administrative database that contained elements of the enrollees’ outpatient medication use and hospitalizations record. The medical record is not currently connected to this medical claims database at the time of the analysis. Hence, specific descriptions of the indication of the medication or what took place in the hospital were not incorporated into this investigation. Further, the analysis performed was an association study that examined the excess likelihood of health services use for users of both agents compared to AChEI users alone. A causal relationship cannot be confirmed in this analysis. Due to the nature of this dual Medicare–Medicaid database, we were not able to adjust for additional potential confounders. Accordingly, this analysis represented an unadjusted exploratory analysis of the relationship. Similarly, aspects of the generalizability of this study are limited based on the absence of the demographic characteristics for the sample.

As the population continues to age in the US, older adults will be more likely to experience both Alzheimer’s disease and overactive bladder at the same time. Thus, possible consideration of using acetylcholinesterase inhibitors for syndromes such as Alzheimer’s disease and anticholinergics for overactive bladder will also rise as will the possible health consequences from these antagonistic therapies. For the patients using acetylcholinesterase inhibitors in particular, symptom control for overactive bladder must emphasize lifestyle modifications as its first-line. For those that require additional therapy, non-anticholinergic oral therapies should be considered such as beta-3 adrenergic agonist drugs such as mirabegron and vibegron, or possibly interventions for those that are considered refractory to oral anticholinergics such as intra-detrusor injections of onabotulinum toxin.

Our findings showed that greater than 10% of users of acetylcholinesterase inhibitors also used anticholinergics for overactive bladder treatment. This was associated with increased hospitalization in the same year. Clinicians should consider improved monitoring of the usage of both medications and clarify the alternative regimens for overactive bladder treatment that avoid anticholinergic agents. To the best of our knowledge, this was the first exploration of the hospitalization risk associated with concomitant use of acetylcholinesterase inhibitors and anticholinergics for overactive bladder in a nationally representative, observational database. Future analyses will include multi-year examinations of this phenomenon and incorporation of additional patient data.

## 5. Conclusions

In the Medicare and Medicare–Medicaid dual-eligible population, concurrent usage of an overactive bladder anticholinergic in acetylcholinesterase inhibitor users exceeded ten percent. These patients were at elevated risk for hospitalization. Further robust, prospective studies are needed for analysis of the phenomena observed in this study. That said, clinicians should consider improved monitoring of the usage of both medications and clarify alternative regimens for overactive bladder treatment that avoid the use of anticholinergic medications in the older adults who also use acetylcholinesterase inhibitors for Alzheimer’s disease.

## Figures and Tables

**Table 1:** Frequency of same-year hospitalization and use of both AChEIs and anticholinergics for overactive bladder in Medicare older adults.

	Hospitalization (*n* = 38,790)	No Hospitalization (*n* = 83,230)	Chi-Squared Test of Association *p*-Value
User of both AChEIs and anticholinergics for overactive bladder	5 608 (35.9)	10 031 (64.1)	<0.001
User of AChEIs alone	33 182 (31.2)	73 199 (68.8)	

**Table 2:** Odds ratios for increased probability of hospitalization for those using both AChEIs and anticholinergics in the same year in Medicare seniors versus Medicare–Medicaid dual-eligible older adults.

	Medicare Older Adults Only	Medicare–Medicaid Dual-Eligible Older Adults
User of both AChEIs and anticholinergics for overactive bladder	1.23 [95% CI, 1.19, 1.28]	1.14 [95% CI, 0.99, 1.31]

**Table 3:** Frequency of same-year hospitalization and use of both AChEIs and anticholinergics for overactive bladder in Medicare–Medicaid dual-eligible older adults.

	Hospitalization (*n* = 2102)	No Hospitalization (*n* = 6813)	Chi-Squared Test of Association *p*-Value
User of both AChEI and anticholinergics for overactive bladder	298 (25.7)	862 (74.3)	0.07
User of AChEs alone	1 804 (23.3)	5 951 (76.7)	

## Data Availability

Data analyzed was from licensed from the MarketScan Claims Databases during the time of research completion and was previously subject to required destruction upon expiration of licensing period.
